# A Question of Professional Conduct

**Published:** 1914-06-20

**Authors:** 


*To the Editor of* The Hospital.

### A Question of Professional Conduct.

*To the Editor of* The Hospital.

Sir,—With regard to my letter which you so kindly inserted in this week's Hospital, I should like to say that when the practitioner in question was asked for an explanation for taking such a step he merely lost his temper and called the husband a cruel man for suggesting that the patient should have been allowed to go on suffering more "unnecessary" pain. The words given in the last letter *re* smoking, &c., were his further comments.

I should be glad if you or some of your readers could say whether chloroform ever has any effect on the milk-glands. Previous to confinement the patient had every prospect of a plentiful milk supply, but after confinement it seemed to have gone away, and the little she had disagreed with the baby, though the patient herself has always been healthy.

I should further esteem it a great favour if you would state whether the practitioner exceeded his legal right if the facts are as stated?—I am, Sir, yours faithfully,

June 12, 1914. X. Y. Z.

[Subject to the conceivable possibility of the practitioner having had in this case some good reason for his action which he did not disclose, we remain of the opinion formerly expressed that his conduct was indefensible, and that his treatment was bad. Chloroform has no such effect on the milk secretion as our correspondent inquires about: we can say confidently that the explanation of the failure of lactation cannot be ascribed to this cause. As for "exceeding his legal right," if that phrase means "Did the doctor lay himself open to a successful action for *malapraxis*?" we should feel inclined to say that he did not, though we are not aware of any similar case ever having been laid before a judge and jury.—Editor, The Hospital.]

